# DNA methylation signature (SAM40) identifies subgroups of the Luminal A breast cancer samples with distinct survival

**DOI:** 10.18632/oncotarget.13718

**Published:** 2016-11-30

**Authors:** Thomas Fleischer, Jovana Klajic, Miriam Ragle Aure, Riku Louhimo, Arne V. Pladsen, Lars Ottestad, Nizar Touleimat, Marko Laakso, Ann Rita Halvorsen, Grethe I. Grenaker Alnæs, Margit L.H. Riis, Åslaug Helland, Sampsa Hautaniemi, Per Eystein Lønning, Bjørn Naume, Anne-Lise Børresen-Dale, Jörg Tost, Vessela N. Kristensen

**Affiliations:** ^1^ Department of Cancer Genetics, Institute for Cancer Research, OUS Radiumhospitalet, Oslo, Norway; ^2^ Department of Clinical Molecular Biology and Laboratory Science (EpiGen), Akershus University hospital, Division of Medicine, Lørenskog, Norway; ^3^ Systems Biology Laboratory, Institute of Biomedicine and Genome-Scale Biology Research Program, University of Helsinki, Finland; ^4^ Laboratory for Epigenetics and Environment, Centre National de Génotypage, CEA – Institut de Génomique, France; ^5^ Department of Surgery, Akershus University Hospital, Lørenskog, Norway; ^6^ Deptartment of Breast and Endocrine Surgery, Oslo University Hospital, Ullevål, Norway; ^7^ Department of Oncology, Oslo University Hospital, The Norwegian Radium Hospital, Oslo, Norway; ^8^ Section of Oncology, Institute of Clinical Science, University of Bergen, Bergen, Norway; ^9^ Department of Oncology, Haukeland University Hospital, Bergen, Norway; ^10^ Cancer Clinic, Oslo University Hospital Radiumhospitalet, Oslo, Norway

**Keywords:** breast cancer, Luminal A, DNA methylation, classification, prognosis

## Abstract

Breast cancer patients with Luminal A disease generally have a good prognosis, but among this patient group are patients with good prognosis that are currently overtreated with adjuvant chemotherapy, and also patients that have a bad prognosis and should be given more aggressive treatment. There is no available method for subclassification of this patient group. Here we present a DNA methylation signature (SAM40) that segregates Luminal A patients based on prognosis, and identify one good prognosis group and one bad prognosis group. The prognostic impact of SAM40 was validated in four independent patient cohorts. Being able to subdivide the Luminal A patients may give the two-sided benefit of identifying one subgroup that may benefit from a more aggressive treatment than what is given today, and importantly, identifying a subgroup that may benefit from less treatment.

## INTRODUCTION

Breast cancer is a heterogeneous disease that is driven by different genetic and epigenetic changes [1, 2]. Epigenetic changes are considered to be an early event in tumor development and one of the hallmarks of cancer. The degree of DNA methylation in the promoter region of tumor suppressor genes, DNA repair genes and transcription factors may play a role in the initiation of cancer, tumor progression and response to treatment [3, 4].

Gene expression profiling classified breast cancers into several molecular subtypes that differ significantly in incidence, survival and response to therapies: Luminal A, Luminal B, HER2 enriched, Basal-like and Normal-like [5–9]. Patients with Luminal A tumors usually have the best prognosis [7]; this holds true also when tumors are treated with contemporary adjuvant chemotherapy including anthracyclines and taxanes [10].

Large scale methylation analyses have shown that breast cancers may also be classified by DNA methylation status, whereby tumors segregate into three clusters. These clusters are associated with overall survival, molecular subtype, ER expression and *TP53* mutation status [11–15]. In a previous study, our group investigated the methylation status in about 800 cancer related genes across a panel of breast cancers and showed that luminal A tumors were quite evenly divided between two of the methylation derived clusters [15].

Using analysis of copy number aberrations (CNA) in tumors, Ciriello and colleagues [16] reported that Luminal A tumors may be separated into four groups characterized by distinct patterns of CNA and different clinical outcome. One subgroup with high level of genomic instability was associated with a poor prognosis (copy number high (CNH) samples) and had molecular features atypical of Luminal tumors.

In the clustering analysis of joint copy number and gene expression data from the cis-associated genes Caldas and colleagues revealed 10 integrative clusters (IntClust 1–10) [17]. Luminal A tumors were divided between three clusters (IntClust 3, 7 and 8); IntClust 3 was associated with the best prognosis with a 10-year of around 90%, while IntClust 7 and 8 showed a 10-year disease-specific survival rates of around 80%.

Expression of miRNAs has also been proposed to influence the methylation profile of cancers as it was demonstrated that for example the miRNA-29 (miR-29) family targets directly *DNMT3A* and *DNMT3B* in lung cancer [18].

The aims of the present study were first to refine and validate the classification of Luminal A breast cancers based on DNA methylation profiles across several datasets and, second, to address the prognostic impact of such classification, in particular aiming at identifying a subgroup of Luminal A tumors with a good prognosis in no need of adjuvant chemotherapy. Third, we investigated how the split of the Luminal A group may be affected by expression of miRNAs of the miR-29 family, and fourth, we investigated to what extent the split of the Luminal A group was affected by DNA copy number.

## RESULTS

### SAM40 – a DNA methylation signature stratifying patients with Luminal A breast tumors according to prognosis

We previously reported that Luminal A tumors were segregated into two different methylation derived clusters based on analysis of 1505 CpGs in 807 genes ([15]; Illumina GoldenGate). Using the Illumina GoldenGate DNA methylation profiles we applied SAM to identify differentially methylated genes. Forty-one genes were found significantly differentially methylated between the two methylation clusters of the Luminal A tumors. The genes were *ADAMTS12*, *ASCL2*, *BIRC4*, *BMP3*, *BMP6*, *CD40*, *CDKN1C*, *COL1A2*, *DES*, *DKC1*, *DLK1*, *EGFR*, *ESR2*, *ETS1*, *ETV1*, *FES*, *FLT4*, *HBII-52*, *HOXA11*, *ICAM1*, *IRAK3*, *KIT*, *KRT13*, *LYN*, *MAS1*, *MKRN3*, *MYBL2*, *PALM2-AKAP2*, *PAX6*, *PCDH1*, *PDGFRB*, *PEG10*, *PITX2*, *SFRP1*, *TERT*, *TMEFF1*, *TNFRSF10C*, *TNFSF8*, *TPEF*, *WNT1* and *WT1*. The methylation status of these genes or any subset available on a given DNA methylation platform is from now referred to as SAM40.

**Figure 1:**
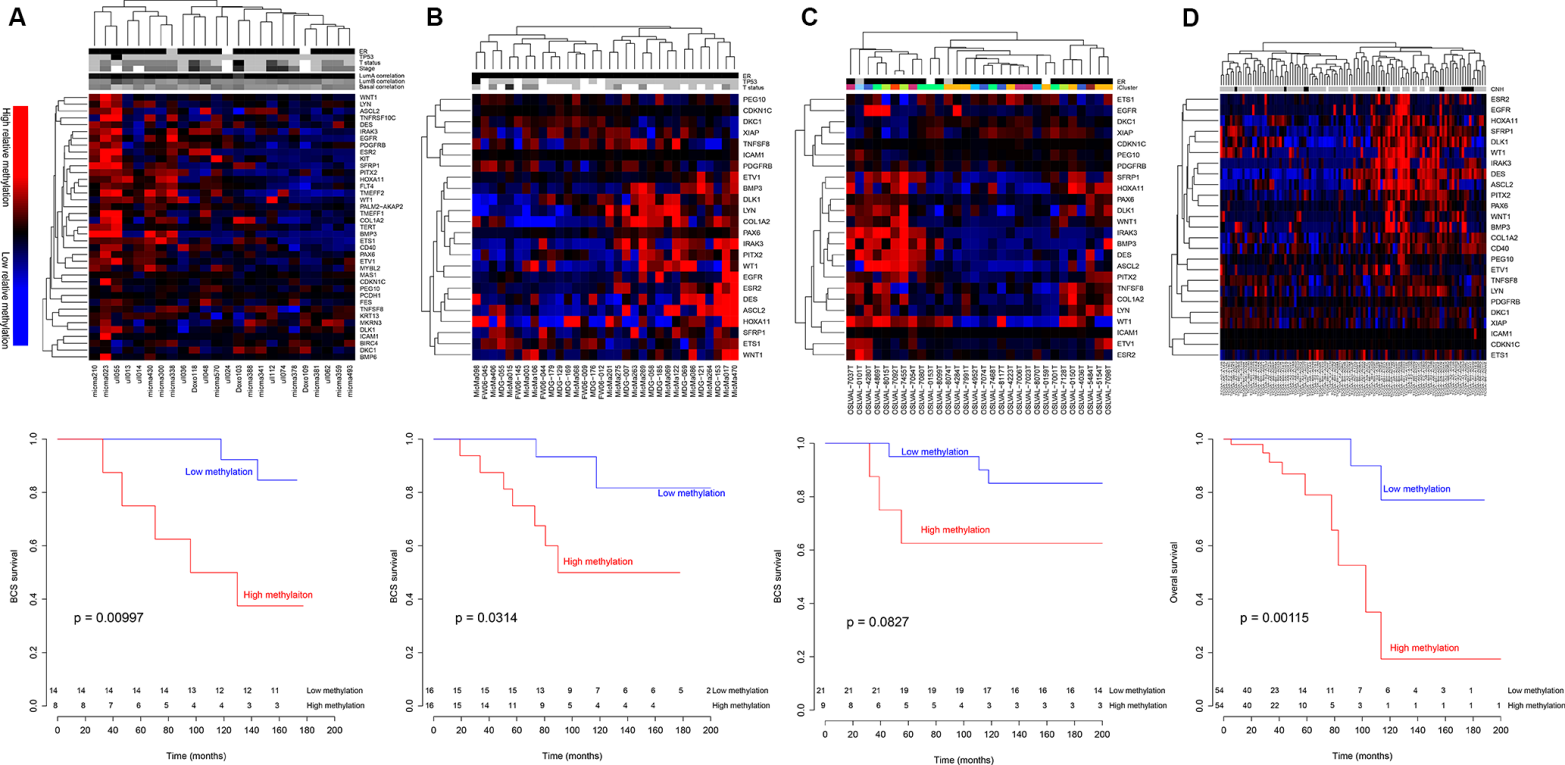
Hierarchical clustering of patients with Luminal A tumors using the SAM40 methylation signature Patients were divided into two groups with different prognosis. The samples were divided in one cluster with low relative methylation and one cluster with high relative methylation (upper panels). The patients with low methylation had better prognosis in all four study cohorts (lower panels). (**A**) Norway27K study cohort (24 samples, 39 genes), (**B**) Norway450K study cohort (32 samples, 24 genes), (**C**) OsloVal (30 samples, 24 genes) and (**D**) TCGA (108 samples, 24 genes). Patient characteristics are indicated: estrogen receptor status: ER negative (gray) and ER positive (black); TP53 mutation status: wild type (gray) and mutated (black); T status: T2 (light gray), T2 (grey), T3 (dark grey) and T4 (black); TNM stage: stage I (light gray), stage II (grey) and stage III (dark grey); correlation to PAM50 centroids: high (dark) and low (light); iCluster: color corresponds to [17]; copy number high classification: non-CNH (gray), CNH (black) and unknown (white) as described by Ciriello et al. [16]. Unknown status is denoted with white.

The identified 41 genes (or the subset available for the different methylation data sets) were used to perform hierarchical clustering of Luminal A tumors in all four study cohorts. In the Norway27K cohort (HumanMethylation27) 39 genes were available (*ADAMTS12* and *HBII-52* did not have probes on the 27K array). The methylation levels of all probes mapped to each gene were summarized using the median. Hierarchical clustering showed that the samples segregated into two clusters, one with relatively high methylation (red) and one with relatively low methylation (blue; Figure 1A upper panel). DNA methylation data for the three remaining study cohorts (Norway450K, OsloVal and TCGA) were generated using the HumanMethylation450. The methylation level of the probes that represented the 5′UTR of each gene was used for hierarchical clustering. The genes that were not found with probes in the 5′UTR were *ADAMTS12*, *BMP6*, *CD40*, *FES*, *FLT4*, *HBII-52*, *KIT*, *KRT13*, *MAS1*, *MKRN3*, *MYBL2*, *PALM2-AKAP2*, *PCDH1*, *TERT*, *TMEFF1*, *TNFRSF10C* and *TPEF*. Like the Norway27K, the three remaining Luminal A study cohorts were each divided in two clusters: one cluster with high relative methylation (red) and one cluster with low relative methylation (blue; Figure 1B–1D upper panels).

Kaplan-Meier analyses and log-rank tests were applied to the four study cohorts to determine the prognostic impact of the SAM40 signature. In all four study cohorts the patients belonging to the cluster with low relative methylation had better prognosis (Figure 1 lower panels). In the Norway27K and Norway450K study cohorts patients in the hypomethylated cluster showed significantly better breast cancer specific survival (*p* = 0.00997 and *p* = 0.0314, respectively), while in the OsloVal study cohort patients in the hypomethylated clusters showed borderline significantly better breast cancer specific survival (*p* = 0.0827). In the OsloVal study cohort, the segregation was statistically significant for overall survival (*p* = 0.0203; Supplementary Figure 1). In the TCGA, patients in the hypomethylated cluster showed significantly better overall survival (*p* = 0.00115).

### Other parameters influencing prognosis of patients

ER status, *TP53* mutation status, TNM stage and correlation to gene expression derived subtype centroids were determined for samples in the Norway27K study cohort. Only one tumor was found to be ER negative and only one tumor harbored a *TP53* mutation. No differences with respect to TNM stage or the distance to the gene expression derived subtype centroids (Luminal A, Luminal B or Basal-like) were observed between tumors classified in the two clusters (Figure 1A upper panel).

Ciriello et al. [16] reported a subgroup of Luminal A tumors with high level of genomic instability associated with a poor prognosis (copy number high (CNH) samples). The CNH samples in the TCGA data were evenly distributed between the two SAM40 derived clusters.

Curtis et al. [17] reported that breast cancer could be divided in ten subgroups based on CNA and gene expression profiles (iCluster). This classification was available for the samples in the OsloVal study cohort, and there was no clear difference in distribution of the iClusters between the two methylation derived clusters (Figure 1C upper panel).

### Absolute methylation levels

Comparing the absolute methylation levels of the SAM40 genes in the two clusters of the Norway 27K cohort revealed 18 out of 39 genes (available on the 27K array) to be methylated at significantly higher levels in the left cluster compared to the right cluster of Figure 1A. Methylation level of normal breast tissue was also compared to methylation level of tumors in the two clusters, and 27 genes were differentially methylated between the hypermethylated cluster and normal tissue, while 21 genes were differentially methylated between the hypomethylated cluster and normal tissue. In general, absolute methylation levels of the samples in the hypomethylated cluster differed from normal controls to a lower extent than samples in the hypermethylated cluster. Absolute methylation level for all genes is shown graphically in Supplementary Figure 2, and *p*-values for statistical comparisons are given in Supplementary Table 3.

### Pathway analysis

Pathway analysis of the SAM40 genes identified significant association to canonical pathways such as BMP, NF-κB, IL-8, PTEN and telomerase signaling, and regulation of the epithelial-mesenchymal transition (EMT) pathway. All significant pathways are shown in Table 1.

**Table 1:** Ingenuity pathway analysis of the 41 genes in the SAM40 signature

	Benjamini Hochberg corrected *p*-value	Ratio	Molecules
Hepatic Fibrosis / Hepatic Stellate Cell Activation	0.00015	0.04	COL1A2,ICAM1,CD40,FLT4,PDGFRB,EGFR
BMP signaling pathway	0.00229	0.05	BMP3,PITX2,BMP6,XIAP
NF-kappaB Signaling	0.00229	0.03	CD40,FLT4,IRAK3,PDGFRB,EGFR
Telomerase Signaling	0.00324	0.04	ETS1,TERT,DKC1,EGFR
Role of Osteoblasts, Osteoclasts and Chondrocytes in Rheumatoid Arthritis	0.00525	0.02	BMP3,SFRP1,BMP6,WNT1,XIAP
Human Embryonic Stem Cell Pluripotency	0.00724	0.03	BMP3,BMP6,WNT1,PDGFRB
Basal Cell Carcinoma Signaling	0.01349	0.04	BMP3,BMP6,WNT1
Regulation of the Epithelial-Mesenchymal Transition Pathway	0.01660	0.02	ETS1,WNT1,PDGFRB,EGFR
IL-8 Signaling	0.01660	0.02	ICAM1,FLT4,IRAK3,EGFR
Role of NANOG in Mammalian Embryonic Stem Cell Pluripotency	0.03311	0.03	BMP3,BMP6,WNT1
PTEN Signaling	0.03631	0.02	FLT4,PDGFRB,EGFR
Atherosclerosis Signaling	0.03631	0.02	COL1A2,ICAM1,CD40

### Multivariate analysis

To investigate whether the SAM40 signature was an independent prognostic marker, a multivariate Cox proportional hazard model was also performed to adjust for therapy regime, age, lymph node status and T status. The analyses were performed in the Norway27K study cohort (Figure 1A; Table 2A) and in the Norway450K study cohort (Figure 1B; Table 2B). Classification by SAM40 was significantly associated with survival in the multivariate model for the samples in the Norway 27K study cohort (*p* = 0.028), and borderline significant for the samples in the Norway450K study cohort (*p* = 0.072). The results are summarized in Table 2.

**Table 2:** Multivariate Cox proportional hazard survival analysis

A				
	Coefficient	Hazard ratio	Standard Error of coefficient	*p*-value
SAM40 signature	3.72	41.16	1.70	0.028
Recieved chemotherapy	−18.4	1.04E–08	2.01E + 04	0.999
Recieved hormonetherapy	−1.89	0.15	1.91	0.322
Age (Older than 55)	1.67	5.32	1.97	0.396
Lymph node positive	3.31	27.49	1.87	0.076
T2 or T3	−0.18	0.83	2.31	0.937

B				
	Coefficient	Hazard ratio	Standard Error of coefficient	*p*-value
SAM40 signature	1.59	4.92	0.89	0.072
Recieved chemotherapy	0.14	1.14	1.60	0.933
Recieved hormonetherapy	−2.31	0.10	1.53	0.131
Age (Older than 55)	1.34	3.81	1.00	0.181
Lymph node positive	2.57	13.1	1.65	0.120
T2 or T3	0.48	1.62	1.12	0.666

Coefficients, hazard ratios, standard error of coefficients and *p*-values are shown for each investigated variable. A) Norway27K study cohort. Samples correspond to those presented in Figure 1 B) Norway450K study cohort. Samples correspond to those presented in Figure 1.

### Analysis of synergistic effects of deregulated methylation and copy-number alteration

To investigate whether the genes in the SAM40 signature were affected concomitantly by methylation and genomic alteration CNAmet analysis was performed on samples of the Luminal A subtype. CNAmet is an algorithm to analyze the simultaneous and synergistic (additive) effect of methylation and copy-number alteration on gene expression in cancer. When the analysis was performed on the SAM40 genes, only two genes (*FES* and *TNFRSF10C*) showed borderline significant synergistic effects (*q* < 0.2, CNAmet score > 0).

### microRNAs that target DNMTs may cause differential methylation patterns in Luminal A tumors

**Figure 2:**
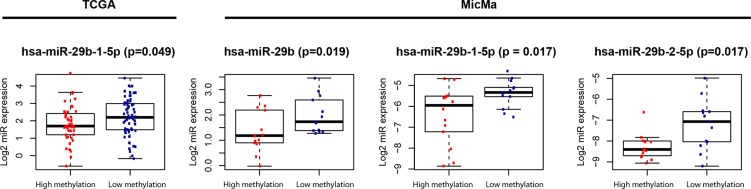
Differential expression of miRNAs in the miR-29 family

Expression of miRNAs in the miR-29 family, known to target the DNA methyltransferases *DNMT3A* and *DNMT3B* was tested for differential expression in the subgroups of Luminal A tumors. In the TCGA study cohort one miRNA (hsa-miR-29b-1-5p; *p* = 0.049) was found higher expressed in the samples belonging to the hypomethylated cluster. When investigating all samples from the MicMa cohort, three miRNAs (hsa-miR-29b, hsa-miR-29b-1-5p and hsa-miR-29b-2-5p; *p* = 0.019, *p* = 0.017 and *p* = 0.017, respectively) were also found with higher expression in samples belonging to the hypomethylated cluster (Figure 2). Correlation between miR-29b expression and DNMT expression was investigated, and expression of hsa-miR-29b was negatively correlated to expression of *DNMT3B* (*p* = 0.057).

## DISCUSSION

Here we report a DNA methylation signature (the SAM40) that segregates patients with Luminal A breast tumors in two groups with different prognosis. This observation was made in four independent data sets including The Cancer Genome Atlas.

It is often difficult for the clinician to assess risk of recurrent disease as well as death of disease, and this poses a major challenge for the field. Overtreatment of breast cancer is a problem in the field, and this problem is especially prominent for patients with Luminal A tumors. Many patients will have a good prognosis without adjuvant systemic treatment such as chemotherapy, and these treatments may severely diminish quality of life and may cause long term side effects [19]. Our SAM40 signature allows subdivision of Luminal A patients and this will give a two-sided benefit: identification of one subgroup that may benefit from a more aggressive treatment than what is given today; and equally importantly, identification of a subgroup that may benefit from less treatment.

A recent randomized clinical trial from the Danish Breast Cancer Cooperative Group (DBCG) showed that patients with Luminal A tumors had comparable 10-year disease-free survival regardless of whether or not they received adjuvant chemotherapy [20]. This highlights the importance of choosing the correct patients for treatment.

A limitation of our study is that each Luminal A study cohort is retrospective and rather small. Future studies of the clinical impact of the SAM40 stratification in larger patient cohorts are of utmost importance, and such studies are planned in our hospital.

Multivariate analyses showed that the association between the SAM40 signature and prognosis of Luminal A patients was independent of age, T status and N status. Studies of larger patient populations are needed for definite conclusions.

The genes in the SAM40 signature were enriched in pathways that are known to be important in cancer, such as NF-κB signaling, telomerase signaling, IL-8 signaling, and regulation of EMT. Embryonic pathway signaling and regulation of EMT has been shown to be commonly deregulated in both metastatic breast cancer cells and embryonic stem cells [21]. The genes in the SAM40 signature are all of remarkable function. Bediaga et al. [11] reported that *CD40* was significantly hypermethylated in Luminal B tumors, a patient group with worse survival than those with Luminal A tumors. Both *EGFR* and *FLT4* have been shown to be silenced by DNA methylation in cancer [22, 23]. *LYN* encodes a tyrosine protein kinase and plays an important role in the regulation of innate and adaptive immune responses, responses to growth factors and cytokines, and also responses to DNA damage and genotoxic agents. Fackler et al. [24] have reported hypermethylation of *LYN* in ER-positive tumors. *SFRP1* and *WNT1* are part of the Wnt pathway, which is important in embryonic development, cell differentiation and proliferation. *WNT1* has been reported to be significantly hypermethylated in ER positive tumors [24]. Taken together, many of the genes in the SAM40 are involved in important pathways and functions that are implicated in cancer.

It has been shown that members of the miR-29 family have binding sites in *DNMT3A* and *DNMT3B*, and these miRNAs may therefore be involved in the regulation of DNA methylation patterns [18]. Here we show that miR-29b is differently expressed between the two clusters of Luminal A patients, potentially contributing to the different methylation pattern. miR-29b is higher expressed in the group with lower methylation, suggesting that this miRNA may inhibit the *de novo* DNMTs resulting in tumors with lower methylation.

Synergistic effects between DNA methylation and copy number alterations were only observed in two of the genes studied here. Ciriello et al. [16] identified a bad prognosis subgroup of Luminal A in the TCGA data based on copy number profiles (copy number high; CNH). In the present study CNH Luminal A samples were distributed quite evenly between the high and the low methylation level groups. Thus, our analyses suggest that the genes in the SAM40 methylation signature are mostly uninfluenced by copy-number changes and that the SAM40 classifier is independent of classification by CNAs.

## CONCLUSIONS

Breast cancer patients with Luminal A tumors were split into two groups using a DNA methylation signature (SAM40), and these patients showed significantly different prognosis. This novel signature was replicated and validated in three independent data sets. Being able to identify a subgroup of Luminal A patients with even better prognosis may have important implications for treatment of breast cancer and may be used as valuable tool for avoiding over-treatment of this patient group.

## MATERIALS AND METHODS

### Patient material

The inclusion criteria for this study was 1) that the sample had expression profiling for PAM50 classification 2) that the sample was classified as Luminal A, 3) that the sample had methylation profiling, and 4) that the patient had at least 10 year clinical follow up. The eligible samples were selected from several patient cohorts from the Oslo region in Norway and the DNA methylation profiles have been generated for using two different Illumina Infinium platforms (HumanMethylation27 and HumanMethylation450). The samples were divided in three study cohorts based on DNA methylation profiling platform and patient characteristics: Norway27K, *n* = 24 [25–27], Norway450K, *n* = 32 [25, 28, 29] and OsloVal, *n* = 30 [30]. In addition, data from TCGA, *n* = 108 were downloaded and comprised the fourth study cohort [12]. The four study cohorts are summarized in Supplementary Table 1 and the patient characteristics are summarized in Supplementary Table 2. Molecular subtypes were determined on each main cohort separately according to the PAM50 classification algorithm [31]. All patients gave their informed consent, and each individual study was approved by the regional ethical committee. Since Luminal A patients generally have a good prognosis, the most appropriate end point for prognosis was breast cancer specific survival (BCSS) and this was used for the three Norwegian study cohorts. For TCGA, only overall survival data (OS) was available.

### DNA methylation analysis

Bisulfite conversion and DNA methylation analysis using the Illumina Infinium HumanMethylation27 and HumanMethylation450 beadchip assays was carried out as previously described [32, 33]. Preprocessing and normalization involved steps of probe filtering, color bias correction, background subtraction and subset quantile normalization as previously described [34].

Level 3 methylation data from TCGA was downloaded from the TCGA data portal (https://tcga-data.nci.nih.gov; [12]). Probes with more than 50% missing values were removed, and further missing values were imputed using the k-nearest neighbor algorithm (R package pamr; *k* = 10). The clinical data from TCGA were downloaded on December 11th, 2013.

### Identification of a DNA methylation signature for segregation of Luminal A tumors

In Rønneberg et al. [15] we discovered that Luminal A tumors clustered in two different groups when using DNA methylation level of about 1505 CpGs in 807 cancer-related genes. In the present study we validate this clustering using differentially methylated genes in two array platforms, HumanMethylation27 and 450K array. We used the *SAM* function, R package *samr* [35] with 100 permutations to identify differentially methylated genes. Methods for choosing probes that represent the identified genes were different for the two array platforms. For the HumanMethylation27 all probes mapped to the each gene were used, and the methylation values for each sample were summarized using the median, resulting in one methylation value per sample per gene. This could be done because the HumanMethylation27 only contains promoter probes. For the HumanMethylation450 a similar approach was used, but using only probes that were mapped to the five prime untranslated regions (5′UTR). Due to differences in coverage of the methylation assays, 39 genes were covered on the HumanMethylation27, and 24 genes had probes in the 5′UTR on the HumanMethylation450.

### CNAmet analysis

CNAmet analysis was carried out in the Anduril workflow environment and with CNAmet version 1.2.1 [36, 37]. Level 1 gene expression and copy-number and level 3 DNA methylation microarray data were downloaded from the Cancer Genome Atlas [12]. A total of 85 Luminal A subtype primary breast carcinoma tumors had all three types of measurement. Copy-number data from Affymetrix 6.0 SNP arrays were extracted with the R package *crlmm* [38]. Samples with signal-to-noise ratio of less than 5 were removed. Moreover, probes with a confidence limit less than 0.9 were removed. Samples were normalized to a mean of 2. Log ratios were segmented using circular binary segmentation (parameters undo.splits=sdundo, undo.SD=3) [39]. All regions where segmented log ratios were over 2.3 were considered copy-number gains and below 1.7 as copy-number deletions. Agilent gene expression microarrays were compared to 59 controls. First, probes matching either multiple or no genes were removed. Then, data were normalized to a mean of 0. The CNAmet analysis was performed using Level 3 Illumina HumanMethylation27 methylation data. The data was preprocessed by TCGA, and in addition, probes with more than 25 missing values were removed.

CNAmet requires binary copy-number and methylation calls. In copy-number data, genes were dichotomized according to their gain/deletion status in a sample. In the methylation data, samples with methylation values in the lowest decile for each gene were considered hypomethylated. Similarly, samples with methylation values in the highest decile for each gene were considered hypermethylated.

Furthermore, the synergistic effect of copy-number gain and hypomethylation, and copy-number deletion and hypermethylation was analyzed separately. Genes with a *q*-value of less than 0.2 and scores over 0 in the CNAmet analysis were considered significant.

### Expression of miR-29 family members and DNA methyltransferases

Expression levels of miRNAs from the miR-29 familiy and mRNA from the DNMTs were available for the MicMa cohort [40] (GEO accession number GSE19536) and the TCGA cohort [12]. As described earlier (Supplementary Table 1), samples from the MicMa cohort were split between two study cohorts due to platform differences when comparing DNA methylation. When comparing expression levels, all MicMa samples were treated as one cohort.

### Statistical analysis

All analyses were performed using the R computing framework [41]. Mean centered beta values were used for hierarchical clustering using Pearson rank correlation matrix in an average linkage clustering approach. Kaplan Meier survival analyses and log-rank tests were performed using the R package *survival*. Multivariate Cox proportional hazard survival analysis was performed using the function *coxph* (R package *survival*) to adjust for treatment regime, age, lymph node status and T status. None of these parameters were significantly associated to survival in univariate analyses, but were included because they are thought to be prognostic in the population. Each parameter in the multivariate model was investigated for violations of the assumption of proportional hazards using the function *cox.zph* (R package *survival*). Data were also analyzed with Ingenuity Pathways Analysis (Ingenuity^®^ Systems, www.ingenuity.com).

## SUPPLEMENTARY MATERIALS


