# Supplementary material for: DNA methylation signature (SAM40) identifies subgroups of the Luminal A breast cancer samples with distinct survival
Source: Oncotarget. 2016 Nov 30;8(1):1074–82. doi: 10.18632/oncotarget.13718 (PMC5352035; doi:10.18632/oncotarget.13718)
Supplement: Supplementary file 1 [file oncotarget-08-1074-s001.pdf]

DNA methylation signature (SAM40) identifies subgroups of the Luminal A breast cancer samples with distinct survival

Supplementary Materials

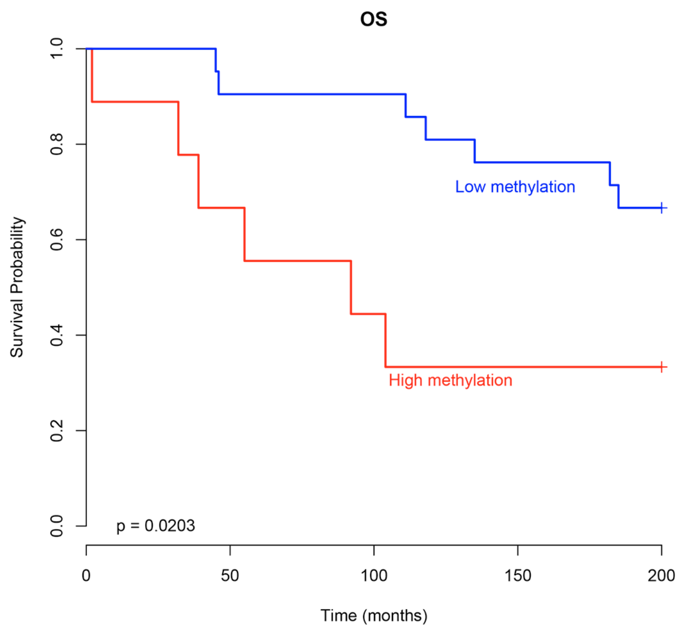

Supplementary Figure S1: Overall survival analysis of the OsloVal study cohort. The prognostic impact of classification using the SAM40 signature is statistically significant.

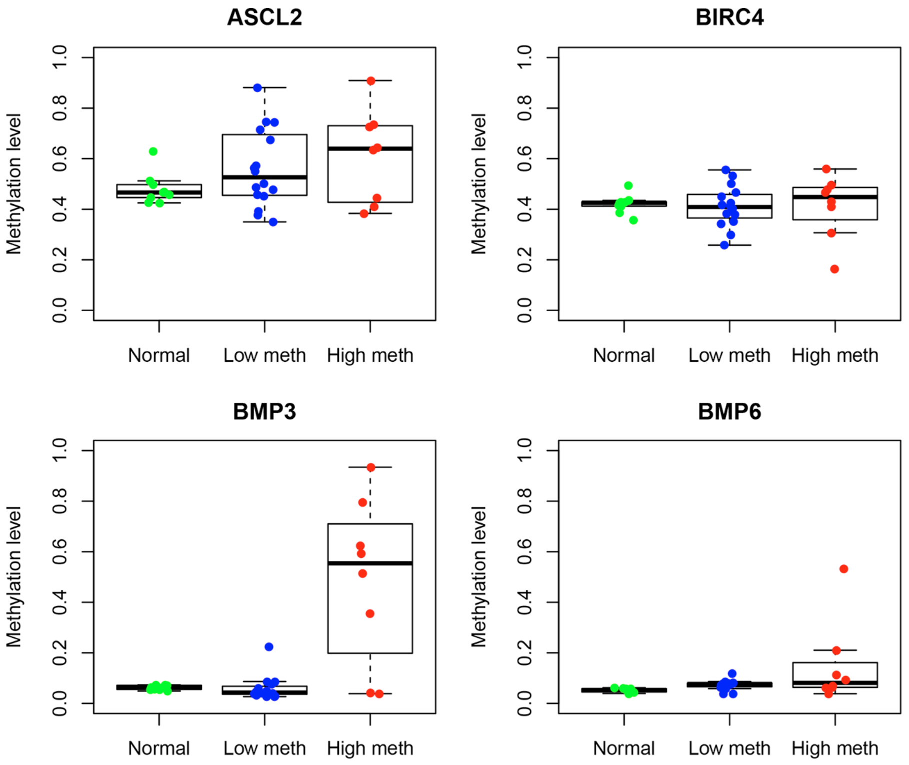

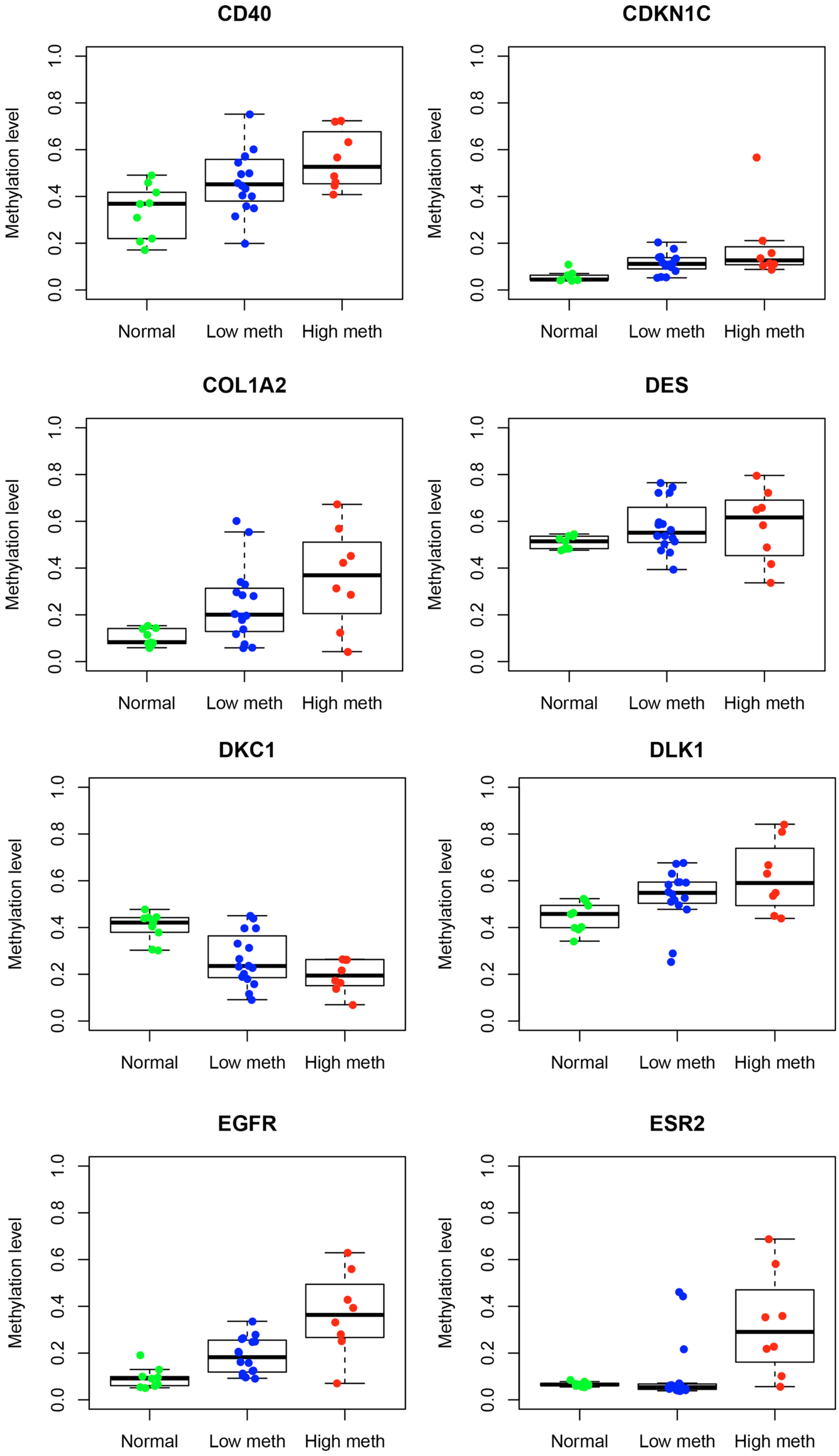

CD40
CDKN1C
COL1A2
DES
DKC1
DLK1
EGFR
ESR2
Methylation level
0.0
0.2
0.4
0.6
0.8
1.0
Normal
Low meth
High meth

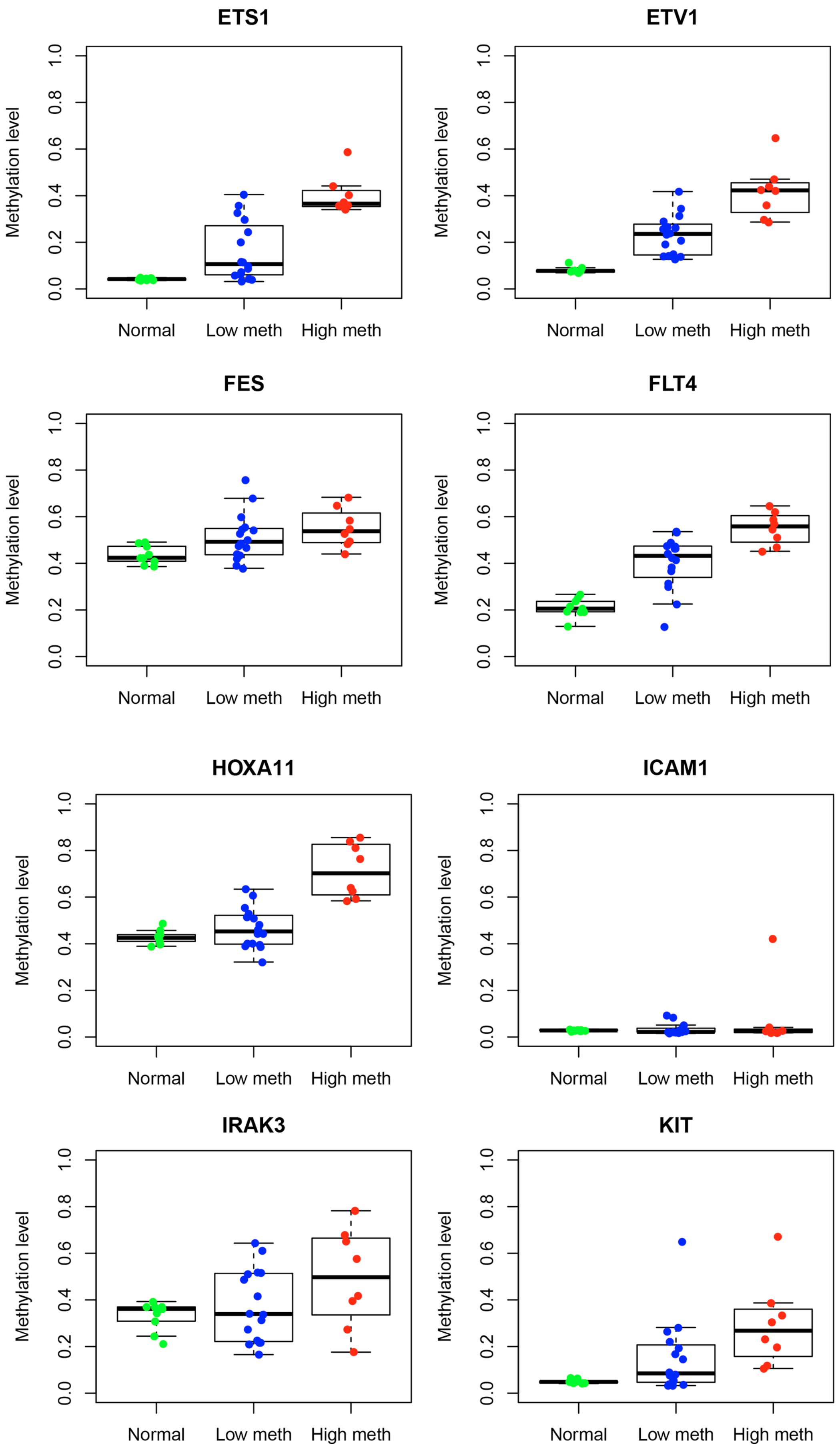
ETS1
ETV1
FES
FLT4
HOXA11
ICAM1
IRAK3
KIT
Methylation level
0.0
0.2
0.4
0.6
0.8
1.0
Normal
Low meth
High meth

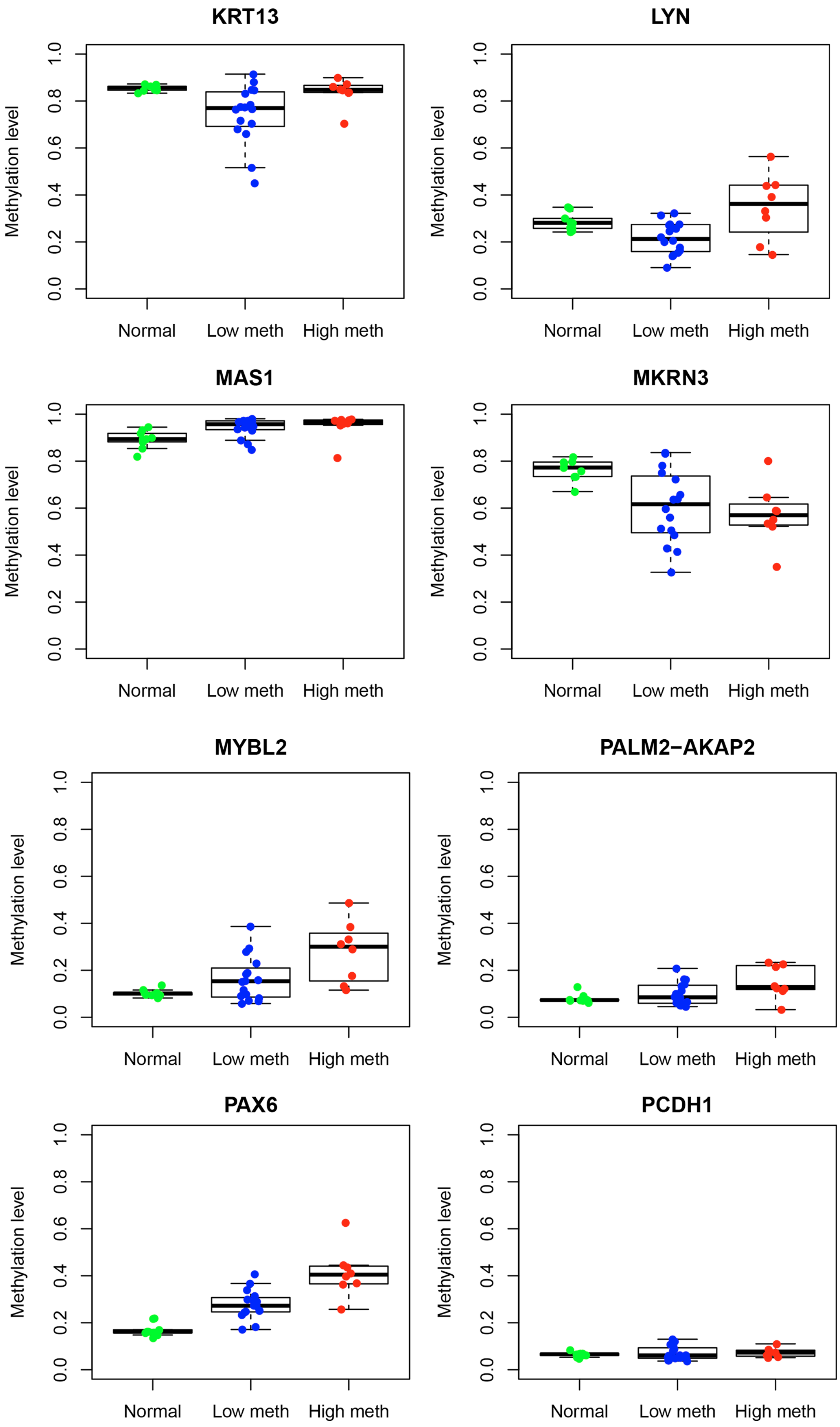
KRT13
LYN
MAS1
MKRN3
MYBL2
PALM2−AKAP2
PAX6
PCDH1
Methylation level
0.0
0.2
0.4
0.6
0.8
1.0
Normal
Low meth
High meth

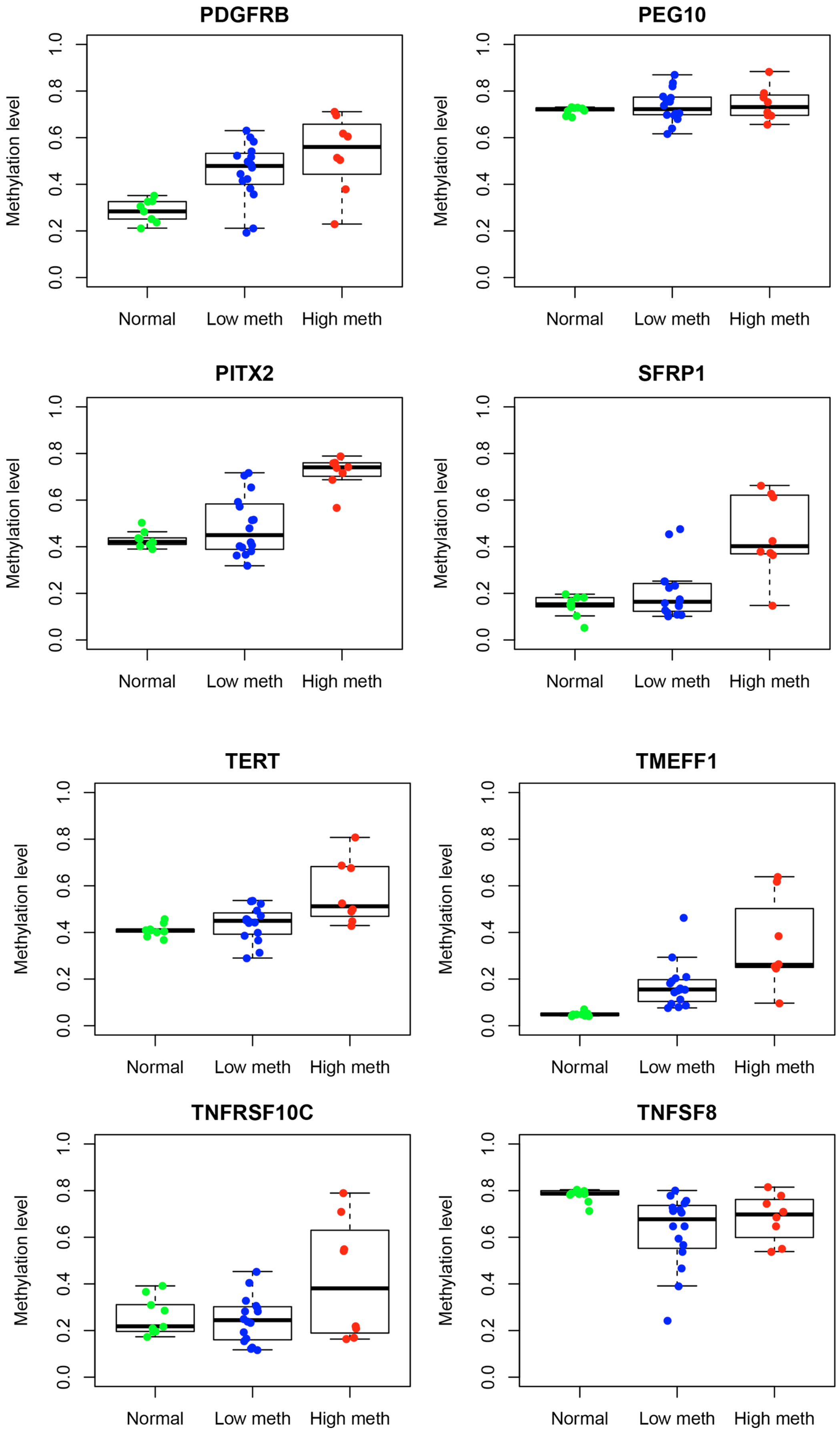
PDGFRB
PEG10
PITX2
SFRP1
TERT
TMEFF1
TNFRSF10C
TNFSF8
Methylation level
0.0
0.2
0.4
0.6
0.8
1.0
Normal
Low meth
High meth

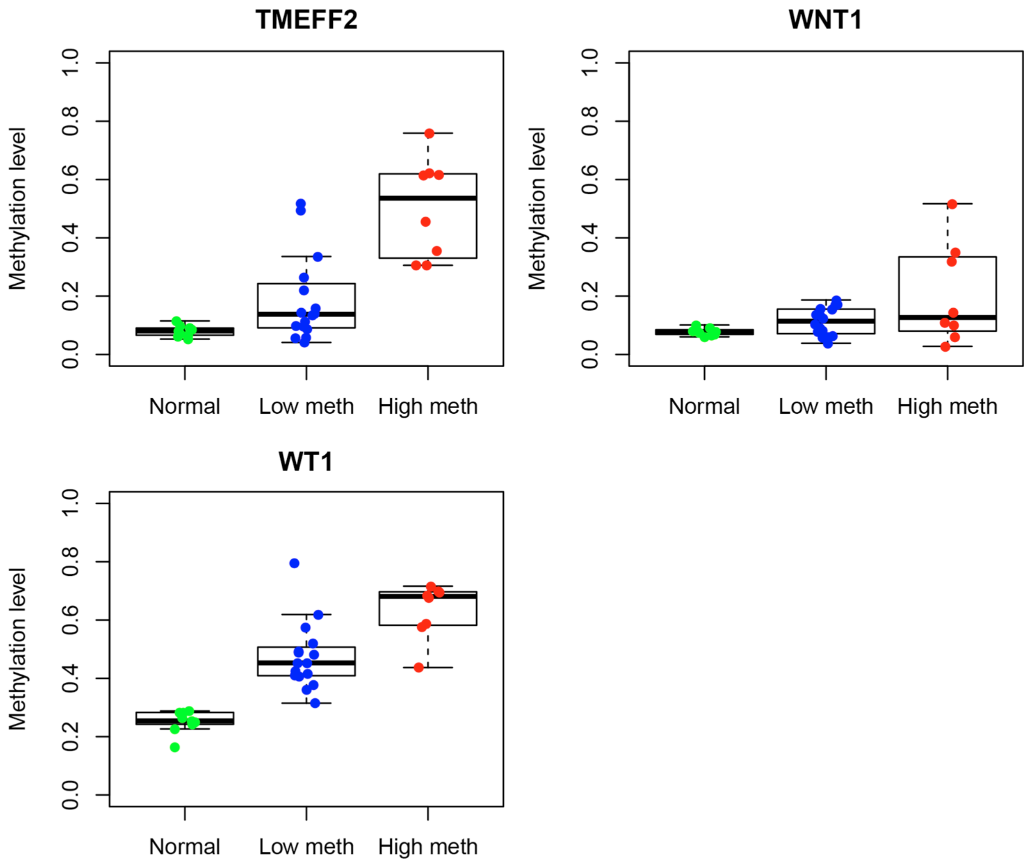

Supplementary Figure S2: Absolute methylation level for genes in the SAM40 signature.

Supplementary Table S1: Material overview

Study cohort	Main cohort	Number of patients	Reference	Ethical approval
	MicMa	12	19	IRB approval no S-97103
Norway27K	Ull	9	20	IRB approval no 285/05
	LB	3	21	IRB approval no 39/92-69.91
	MicMa	16	19	IRB approval no S-97103
Noway450K	FW06	5	22	IRB approval no Dnr 2005:118
	MDG	11	23	IRB approval no S-02036
OsloVal	OsloVal	30	24	IRB approval no 2010/498
TCGA	TCGA	108	13	Not relevant

Supplementary Table S2: Patient characteristics for all four study cohorts

	Norway27K n = 24	Norway450K n = 32	OsloVal n = 30	TCGA n = 108
**Age (median)**	59	71	60	58
**Age (range)**	43–90	36–82	33–85	27–83
**Age unknown**	3	11	0	0
**ER negative**	2	0	2	6
**ER positive**	21	32	26	102
**ER unknown**	1	0	2	0
**Her2 negative**	10	29	0	38
**Her2 positive**	2	1	0	2
**Her2 unknown**	12	2	30	68
**Nstatus negative**	10	7	9	47
**Nstatus positive**	13	7	0	58
**Nstatus unknown**	1	18	4	3
**PAM50 Luminal A**	24	32	30	108
**PAM50 Luminal B**	0	0	0	0
**PAM50 Her2 enriched**	0	0	0	0
**PAM50 Basal-like**	0	0	0	0
**PAM50 Normal-like**	0	0	0	0
**PAM50 unknown**	0	0	0	0
**TP53 wild type**	23	26	0	0
**TP53 mutated**	1	1	0	0
**TP53 unknown**	0	5	30	108
**Tstatus 1**	9	8	14	31
**Tstatus 2**	10	6	10	58
**Tstatus 3**	2	0	2	16
**Tstatus 4**	1	1	2	2
**Tstatus unknown**	2	17	2	1
**Received adjuvant chemotherapy**	8	2	6	30
**No adjuvant chemotherapy**	16	19	18	38
**Unknown adjuvant chemotherapy**	0	11	6	40
**Received adjuvant hormone therapy**	12	10	6	39
**No adjuvant hormone therapy**	12	11	18	29
**Unknown adjuvant hormone therapy**	0	11	6	40
**Received neoadjuvant chemotherapy**	3	0	0	0
**No neoadjuvant chemotherapy**	21	32	30	108
**Unknown neoadjuvant chemotherapy**	0	0	0	0

Supplementary Table S3: Statistical comparisons of absolute methylation values for genes in the SAM40

	p-value		
**Gene**	**Hyper vs Hypo**	**Hypo vs Normal**	**Hyper vs Normal**
ASCL2	6.5E-01	2.3E-01	3.2E-01
BIRC4	5.3E-01	5.2E-01	5.4E-01
BMP3	7.1E-03	7.4E-02	9.3E-02
BMP6	4.9E-01	2.2E-03	7.9E-03
CD40	1.4E-01	3.7E-02	3.7E-03
CDKN1C	2.1E-01	4.7E-04	3.3E-04
COL1A2	1.9E-01	1.7E-02	2.1E-02
DES	9.8E-01	8.4E-02	2.4E-01
DKC1	1.7E-01	5.3E-03	8.2E-05
DLK1	4.2E-01	8.0E-03	1.1E-02
EGFR	8.7E-03	1.4E-03	1.6E-03
ESR2	3.6E-03	1.5E-01	5.5E-03
ETS1	2.6E-04	1.1E-03	8.2E-05
ETV1	1.8E-04	9.8E-07	8.2E-05
FES	2.9E-01	4.3E-02	1.6E-03
FLT4	1.3E-03	2.6E-04	8.2E-05
HOXA11	5.2E-05	3.3E-01	8.2E-05
ICAM1	8.8E-01	2.5E-01	2.4E-01
IRAK3	1.5E-01	8.0E-01	5.9E-02
KIT	1.3E-02	7.4E-02	8.2E-05
KRT13	3.8E-02	5.3E-03	6.7E-01
LYN	1.6E-02	1.4E-02	2.0E-01
MAS1	3.5E-01	6.5E-03	7.9E-03
MKRN3	7.0E-01	9.6E-03	5.5E-03
MYBL2	3.8E-02	2.3E-01	5.8E-04
PALM2-AKAP2	1.1E-01	8.0E-01	2.1E-02
PAX6	9.4E-04	1.2E-05	8.2E-05
PCDH1	4.2E-01	7.2E-01	3.2E-01
PDGFRB	1.7E-01	1.4E-03	5.5E-03
PEG10	9.3E-01	6.0E-01	6.1E-01
PITX2	1.8E-04	7.2E-01	8.2E-05
SFRP1	3.6E-03	4.5E-01	2.5E-03
TERT	2.7E-02	1.5E-01	5.8E-04
TMEFF1	7.1E-03	9.8E-07	8.2E-05
TNFRSF10C	2.1E-01	6.4E-01	4.2E-01
TNFSF8	6.1E-01	6.3E-04	2.1E-02
TMEFF2	3.7E-04	2.7E-02	8.2E-05
WNT1	4.9E-01	9.5E-02	9.3E-02
WT1	3.6E-03	9.8E-07	8.2E-05
#*p*-values < 0.05	18	21	27
